# Bone Mineral Density, Parathyroid Hormone**,** and Vitamin D **A**fter Gastric Bypass Surgery: a 10-Year Longitudinal Follow-Up

**DOI:** 10.1007/s11695-020-04912-7

**Published:** 2020-08-28

**Authors:** Mustafa Raoof, Ingmar Näslund, Eva Rask, Eva Szabo

**Affiliations:** 1 Department of Surgery, Faculty of Medicine and Health, Örebro University, Örebro, Sweden; 2 Department of Medicine, Faculty of Medicine and Health, Örebro University, Örebro, Sweden

**Keywords:** Bone mineral density, Gastric bypass, BMI, Parathyroid hormone, Obesity, Vitamin D, Bone health

## Abstract

**Background:**

The aim of the present study was to study longitudinal changes in bone mineral density (BMD), vitamin D, and parathyroid hormone (PTH) levels in females over a 10-year period after laparoscopic Roux-en-Y gastric bypass (LRYGB).

**Methods:**

Twenty-three women, mean age 43.4 *±* 8.7 years and mean body mass index (BMI) 44.6 ± 5.17 kg/m^2^ at baseline, were included. BMD, BMI, S-calcium, S-25(OH)-vitamin D, and fP-PTH were measured preoperatively and 2, 5, and 10 years postoperatively.

**Results:**

Ten years after surgery, BMD of the spine and femoral neck decreased by 20% and 25%, respectively. Changes in serum levels of vitamin D, PTH, and calcium over the same period were small.

**Conclusion:**

After LRYGB with subsequent massive weight loss, a large decrease in BMD of the spine and femoral neck was seen over a 10-year postoperative period. The fall in BMD largely occurred over the first 5 years after surgery.

## Introduction

Obesity continues to increase in developed countries and even more so in developing countries. In Europe, the estimated prevalence of obesity among adults is 25%, and in Sweden the prevalence among women and men has reached 20% and 24% respectively [1]. Obesity is strongly associated with morbidity and mortality [2, 3]. Surgery is the most effective treatment for obesity, resulting in sustained weight loss [4, 5]. Gastric bypass is a commonly performed procedure worldwide, including Sweden [6, 7]. Though obesity constitutes a considerable health threat, these patients have increased bone mineral density compared with persons with normal weight status [8]. Bariatric procedures, in particular those based on malabsorption, lead to a fall in bone mineral density (BMD) [9–12], an increase in bone resorption and bone remodeling, and changes in bone histomorphometry parameters [10, 13, 14]. The risk for fracture increases at several skeletal sites after bariatric surgery [15–17]. These changes in bone metabolism seem not to be the result of weight loss alone, since BMD continues to decline even when the patient’s weight has stabilized [18]. Vitamin D deficiency [19–23] and secondary hyperparathyroidism [24, 25] could be important in this context; however, the long-term effects of gastric bypass surgery on the skeleton remain unclear.

The aim of this prospective study was to follow a group of female patients longitudinally over a 10-year period after laparoscopic Roux-en-Y gastric bypass (LRYGB) in order to detect changes in BMD and associated changes in serum levels of calcium, vitamin D, and PTH.

## Methods

**Fig. 1:**
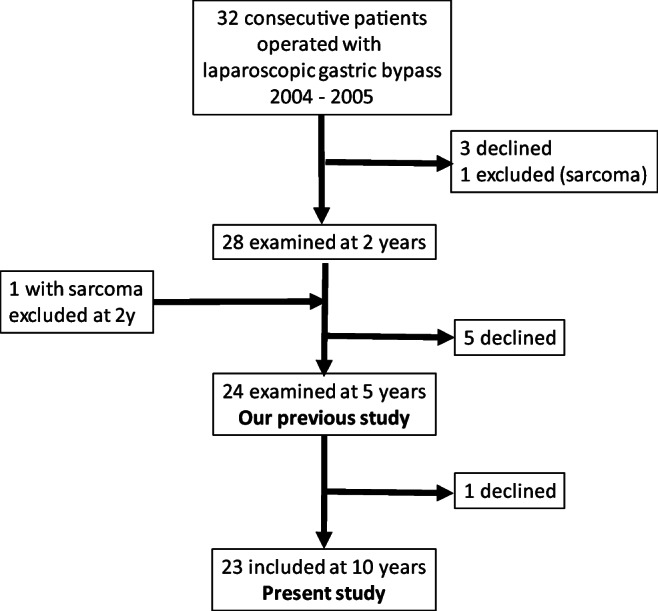
Flow chart of the present and previous study [18]

From January 2004 to December 2005, thirty-two consecutive female patients undergoing LRYGB at the Department of Surgery of the University Hospital of Örebro were recruited for this prospective longitudinal study. This cohort, as well as inclusion and exclusion criteria, has been described previously [18, 26]. Nine patients declined participation in the 10-year follow-up (details in the flow chart, Fig. 1) leaving the study group of 23 women.

BMD and total body fat (% of body weight) were measured using dual-energy X-ray absorptiometry **(**DXA) (DPX-L, Lunar Corp. Madison, WI, USA) preoperatively and at 2, 5, and 10 years postoperatively. Total body, lumbar spine, and femoral neck BMD were recorded. Osteopenia was defined as a BMD between 1.0 SD above and 2.5 SD below the young adult reference mean for women (*t* score), and osteoporosis as a BMD (*t* score) lower than − 2.5 SD.

S-calcium, S-albumin, S-creatinine, fP-alkaline phosphatase, fasting-P-PTH, and S-25-(OH)-vitamin D were measured at baseline, 5, and 10 years postoperatively. The blood samples were analyzed at the local laboratory in accordance with the stipulated routine for each method. Fasting-P-PTH was measured by immunochemistry. Initially, this was calibrated for the normal range of 10–73 ng/L, but during the study period units were changed from ng/L to pmol/L. To convert the results of fP-PTH in pmol/L to ng/L units, we used the equation (X pmol/L)/0.106 [27]. Vitamin D status was based on the level of S-25-OH-vitamin D using HPLC–APCI-MS. Vitamin D deficiency was defined as a S-25-OH-vitamin D < 50 nmol/L [28]. Lab tests and DXA measurements were not done on the same day and a few patients did not turn up the second day, as indicated in Table 3.

## Statistical Analysis

Statistical analyses were performed with IBM SPSS Statistics 23 (IBM, Armonk, NY USA). Unless stated otherwise, continuous variables were presented as mean ± standard deviation. Normality of the continuous variables was evaluated using Shapiro–Wilk test. Standard mean difference and *t* test were used to compare independent groups. Differences of continuous variables between two dependent groups were determined using paired sample *t* test. All tests were two-sided and a *p* value less than 0.05 was considered statistically significant.

## Results

Mean age at baseline was 43.4 ± 8.7 years. The mean preoperative weight was 122.8 ± 14.8 kg, and BMI 44.6 ± 5.17 kg/m^2^. Three patients were treated for diabetes mellitus and one patient had CPAP treatment for sleep apnea syndrome. At 10 years, four patients had treatment for hypertension but no patient was taking medication for diabetes mellitus and no patient required CPAP. The number of menopausal patients increased during the study (Table 1).

**Table 1:** Clinical characteristics of study group

	Preop.	5 years	10 years
Age, years (SD)	43.4 (8.78)		
BMI, kg/cm^2^ (SD)	44.6 (5.17)	31.8 (5.45)	33.0 (4.79)
Height, cm (SD)	165.7 (7.0)	165.5 (7.2)	163.7 (7.2)
DM-drug treatment	3	0	0
HT-drug treatment, *n*	0	1	4
Sleep apnea syndrome, *n*	1	0	0
Menopause, *n*	6		20

As expected, a significant decrease in BMI seen at 5 years (12.6 ± 6.14 BMI-units) and at 10 years (11.6 ± 5.75 BMI-units) were noted compared with baseline. The total body fat percentage decreased by 14% (Table 2).

**Table 2:** Longitudinal DXA and blood chemistry measurements

	Preop.		2 years		5 years		10 years		Paired *t* test		
	Mean	SD	Mean	SD	Mean	SD	Mean	SD	Preop.	5 years	Preop.
									vs 5 years	vs 10 years	vs 10 years
BMD spine, g/cm^2^	1.37	0.13	1.23	0.15	1.12	0.15	1.10	0.45	0.000	ns	0.011
BMD femoral neck, g/cm^2^	1.21	0.11	1.04	0.13	0.91	0.24	0.90	0.14	0.000	ns	0.000
Total bone *t* score	2.84	4.67	0.66	1.04			0.06	1.43			0.011
Spine *t* score	1.43	0.96	0.23	1.23	− 0.48	1.22	− 0.39	1.12	0.000	ns	0.000
Femoral neck *t* score	1.74	1.03	0.30	1.15	− 0.38	1.17	− 0.88	1.21	0.000	0.000	0.000
Total body fat (%)	52.5	4.70			44.9	6.95	45.3	5.30	0.001	ns	0.000
fP-PTH, ng/L *	64.5	19.8			84.8	30.8	57.4	15.4	0.002	ns	0.006
Vitamin D, nmol/L**	45.6	15.2			44.1	21.1	57.0	21.7	ns	0.004	ns (0,050)
Ca corr. for alb., mmol/L***	2.28	0.09	2.19	0.07	2.26	0.07	2.29	0.08	ns	ns	ns

*Ns* not sig (*p* ≥ 0.05)

*= ref.: 10–73 ng/L ** = ref. 75–250 nmol/L *** = ref. 2.15–2.50 mmol/L

**Fig. 2:**
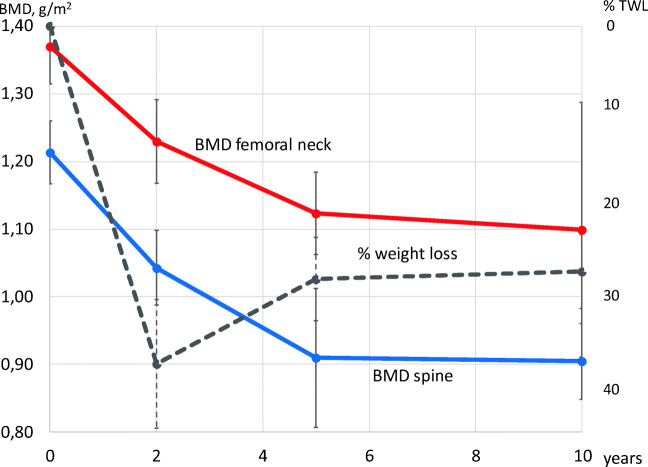
BMD in the spine and the femoral neck and weight loss (% of preop. weight, %TWL) over 10 years; mean ± 95-%-confidence interval

Twenty-two of the 23 patients had initially normal BMD values in both spine and hip (exceeding 1 S. D above reference level in 18), but one woman had a spine DXA measurement showing osteopenia. All measurements showed a fall in bone mineral density over time with statistically significant differences at 5 and 10 years compared with baseline. The fall was greatest during the first 5 years with no significant difference in BMD between 5 and 10 years (Table 2 and Fig. 2). This corresponded to an overall decrease in spine BMD of 20% (0.27 g/cm^2^) and 25% in the femoral neck BMD (0.31 g/cm^2^). One patient had osteopenia prior to surgery and eight patients had developed osteopenia and one osteoporosis in the femur and/or spine at 5 years. DXA measurements at 10 years were basically the same apart from one additional patient who had developed osteopenia, giving nine patients with osteopenia and one with osteoporosis. Three patients with osteopenia and one with osteoporosis were affected in both the spine and the femoral neck.

The mean height of the patients decreased over the 10 years by 1.9 ± 1.05 cm (95% CI: 1.45–2.37, *p* = 0.000).

No patient was admitted or treated for a fracture during the first 5-year period. Between 5 and 10 years, three patients suffered a fracture of the wrist after minor trauma and one of the knee in a bicycle accident. Two of these had osteopenia.

Preoperative vitamin D levels were below 50 nmol/L (deficiency) in 50%, in 12 of 19 at 5 years and in 7 of 21 at 10 years. When patients without (*n* = 15) and those with (*n* = 8) vitamin D/calcium supplementation were compared, no statistically significant differences between preoperative and 10 year variables were observed (Table 3).

**Table 3:** Comparison of patients with and without vitamin D/calcium supplementation at 10 years

	Without vit. D suppl.			With vit. D suppl.				
	*n*	Mean	SD	*n*	Mean	SD	*p* value	smd
Age preop.	15	43.1	7.90	8	44.0	10.82	0.828	0.09
BMI preop.	15	45.4	3.99	8	43.1	6.96	0.328	0.40
BMI 10 y	14	34.2	5.43	8	31.1	3.16	0.156	0.70
Total bone *t* score, 0 year	15	1.953	0.798	8	1.800	0.637	0.645	0.21
Total bone *t* score, 10 years	14	0.007	1.110	8	0.163	1.947	0.813	0.10
Spine *t* score, 0 year	15	1.260	0.803	8	1.738	1.190	0.264	0.47
Spine *t* score, 10 years	15	− 0.647	1.033	8	0.100	1.194	0.132	0.67
Femoral neck *t* score, 0 year	15	1.640	0.931	8	1.925	1.244	0.540	0.26
Femoral neck *t* score, 10 years	15	− 0.940	1.049	8	− 0.763	1.551	0.747	0.13
BMD spine, 0 year	14	1.341	0.122	8	1.419	0.144	0.194	0.58
BMD spine, 10 years	15	0.983	0.396	7	1.348	0.485	0.075	0.82
BMD femoral neck, 0 year	14	1.209	0.092	8	1.220	0.146	0.823	0.09
BMD femoral neck, 10 years	15	0.886	0.121	8	0.940	0.168	0.377	0.37
Total body fat (%), preop	15	53	3.5	8	51	6.3	0.220	0.50
Total body fat (%), 10 years	14	46	4.4	8	44	6.7	0.372	0.38
Vitamin D, preop, nmol/l	15	48.4	13.7	7	39.7	17.7	0.220	0.55
Vitamin D, 10 years, nmol/L	14	53.8	24.7	7	63.3	13.4	0.357	0.48
PTH, preop, ng/L	15	62.3	22.57	8	68.2	13.6	0.481	0.21
PTH, 10 years, ng/L	14	53.2	15.6	7	65.9	12.1	0.076	0.91

Patients not prescribed vitamin D/calcium had a mean vitamin D level that was higher at 10 years than preoperatively, but less pronounced than for the group with supplementation (Table 3).

Patients who declined participation in the 10-year follow-up did not differ significantly in age, comorbidity, or in preoperative measurements from those who did. For ethical reasons, we could not collect information from the medical charts of those patients, making further comparisons impossible.

## Discussion

The main result of this study was the large decrease in bone mineral density seen over 10 years after gastric bypass. This has previously been reported in several studies with follow-up over a few years [29–31] and by us in a longitudinal study over 5 years [18]. We now report on a longitudinal follow-up of the same group of patients (less one) over 10 years. BMD fell throughout the study period albeit at a much slower rate over the last 5 years (no significant difference). Some of the BMD decrease, especially during the first few years during weight loss, could be a result from adaptation to the decreased stress of a heavy body. However, BMD continued to fall even after body weight nadir*.* Our results are in agreement with a recent cross-sectional study from Norway [32] of 124 patients 10 years after gastric bypass. There is also a small study from Denmark with longitudinal data over 7 years reporting similar results [33]. The fall in BMD in these studies was far more pronounced than natural loss of roughly 1% per year in the normal population [12, 34]. In the present study, BMD fell by 20% in the spine and 25% in the femoral neck over the 10-year follow-up. A similar disparity in fall in BMD between spine and femur/hip has been reported by others [35]. However, the fall in spine BMD could be an underestimation since a mean decrease in height of almost 2 cm was observed. This suggests a reduction in vertebral volume (i.e., height) due to compression secondary to a fall in BMD, but could also be caused by soft tissue (discs) reductions.

These findings indicate that following gastric bypass, BMD decreases from almost supernormal to levels approaching osteopenia and osteoporosis, with an increase in fracture risk compared with the general population [16], obese controls, and patients having a restrictive procedure. Two studies compared the gastric bypass procedure with a restrictive procedure such as gastric banding. In these, bypass seemed to carry a greater risk for osteoporotic fractures such as hip fractures, as well as wrist fractures [15, 17]. Reduction in bone mineral density does not fully explain the occurrence of wrist fractures; other risk factors could be involved including lower grip strength, increased walking speed, and increased risk for a fall outdoors [36–38]. Furthermore, decrease in weight is not only loss of fat mass but also lean mass and muscle mass [39]. However, our study was not sized to use fracture as the primary end-point.

At the time our patients were operated (2004–2005), vitamin D/calcium supplementation was not routinely prescribed postoperatively. European recommendations for such supplementation came several years later [40] and formal guidelines for the Nordic countries were published as late as 2017 [41, 42]. Awareness of these recommendations in Swedish primary healthcare has slowly grown over the last years and this is reflected in the number of patients prescribed vitamin D/calcium outside our study. This study was not designed to compare groups with or without vitamin D supplementation, such a study would need larger groups of patients, probably larger doses of supplementation [43], and well-controlled compliance.

Even though low vitamin D and high PTH levels have been reported after gastric bypass [23], it is also known that obesity itself is associated with increased PTH levels as well as low levels of vitamin D. In the present study, half of the patients had vitamin D deficiency preoperatively and a similar number was seen at 10 years in the non-supplemented group. Elevated PTH levels were seen in approximately 25% of patients preoperatively and this remained at 10 years. Considering the great SD for PTH values, we cannot draw certain conclusions from the differences in mean values at the different time points. The changes in PTH do not seem to explain the considerable changes in BMD. Present guidelines have focused on vitamin D/calcium supplementation, but there could well be physiological reasons for the fall in BMD other than lack of vitamin D and secondary hyperparathyroidism, such as changes in gastrointestinal peptides and hormone levels and uptake of protein and other nutrients [44]. In fact, current guidelines are not evidence-based but rather the opinion of experts. We could only find one randomized controlled study addressing this subject in which vitamin D, calcium, and protein supplementation together with physical exercise modified the fall in BMD but did not prevent it [45].

Estrogen deficiency is a well-known cause of low BMD. The mean age at the beginning of this study was 43 years and all but six patients were premenopausal. Obesity is commonly associated with menstrual irregularity leading to relative estrogen deficiency with androgen excess [46], and BMD is usually above normal preoperatively [47]. Bariatric surgery reverses the situation and can lead to resumption of a normal menstrual cycle [48]. It seems unlikely, therefore, that estrogen deficiency explains the decrease in BMD, but measured hormone levels during our study could have been of value.

IGF-1 is the main mediator of the anabolic effects of growth hormone, promoting cell proliferation and growth in several organs, including bone, which correlates with serum insulin levels. The synthesis of IGF-1 is dependent on adequate nutrition, but is also found to be inversely correlated to fat mass [49], more specifically to abdominal fat [50]. The GH/IGF-1-axis is more or less restored after gastric bypass surgery [50, 51] implying that weight loss potentially improves the impaired GH/IGF-1 axis seen in obesity. If, however, this is not accompanied by adequate nutrition, then the positive effect of improved IGF-1 levels may be hampered.

This longitudinal study following gastric bypass is the first to reporting on long-term changes in BMD over a 10-year period. The main limitations are the small cohort size, the spontaneous and uncontrolled consumption of vitamin D during follow-up and lack of an obese control group to compare with. DXA measurement of other parts of the skeleton, as well as more sophisticated laboratory tests measuring bone metabolism, could have provided a broader picture. The two sites we chose, however, are those recommended when diagnosing osteopenia and osteoporosis, and those closely associated with fracture risk. Considering the large changes in BMD observed, it seems unlikely that use of a more advanced DXA-technique measuring volume-BMD instead of area-BMD used here would have had an impact on the main result.

The marked and continued fall in BMD seen in this longitudinal study is probably the consequence an array of factors the nature of which remains unknown. Well-designed interventional studies are required if we are to develop strategies aimed at preventing a fall in BMD after gastric bypass.
